# Peer-led examination simulation reshapes psychophysiological and emotional state profiles during problem-based medical learning

**DOI:** 10.1038/s41539-026-00459-x

**Published:** 2026-10-24

**Authors:** Morris Gellisch, Thorsten Schäfer

**Affiliations:** Center for Medical Education, Ruhr-University Bochum, Bochum, Germany

**Keywords:** Neuroscience, Psychology, Psychology

## Abstract

Repeated problem-based learning (PBL) sessions are intended to sustain active, collaborative, and self-directed learning, yet evidence is mixed as to whether familiar PBL routines consistently maintain an appropriate level of learner activation over time. This repeated-measures study examined whether embedding a peer-led examination simulation into regular PBL reshapes psychophysiological and achievement-emotional state profiles. Forty-two medical students were assessed during resting reference, regular PBL, and PBL with peer-led examination simulation. Heart rate variability (HRV) was quantified using RMSSD, SDNN, pNN50, and LF/HF, alongside anxiety, perceived stress, achievement emotions, motivation, self-rated performance, and post-session appraisal. Both PBL formats reduced HRV relative to rest, indicating autonomic activation during regular PBL itself. Compared with regular PBL, examination simulation produced additional HRV withdrawal, higher anxiety and perceived stress, and lower boredom. Baseline-adjusted mixed models supported effects for RMSSD and SDNN; pNN50 did not survive false-discovery-rate correction, and LF/HF showed no robust effect. Enjoyment, motivation, and self-rated performance did not differ significantly. Students reported greater perceived benefit from and stronger preference for the simulation format. Peer-led examination simulation thus shifted PBL toward a more salient, assessment-like psychophysiological and emotional state profile.

## Introduction

Problem-based learning (PBL) is an established instructional approach in medical education that organizes learning around authentic, complex problems rather than around the passive reception of discipline-based content^1,2^. In PBL, students typically work in small groups to activate prior knowledge, identify knowledge gaps, formulate learning objectives, engage in self-directed inquiry, and collaboratively integrate new knowledge into a shared understanding of the problem^3^. Originating from curriculum reform in medical education, most prominently at McMaster University, PBL was developed to bring learning closer to the intellectual demands of clinical reasoning and professional practice^4,5^. Its enduring appeal lies in a simple but powerful premise: medical knowledge is learned most meaningfully when it is actively constructed, negotiated, and applied in context. Conceptually, PBL builds on constructive, self-directed, collaborative, and contextual learning principles and is therefore considered particularly suited to strengthening clinical reasoning, problem-solving, and professional competencies^6,7^. In many medical curricula, however, PBL is not necessarily implemented as the sole organizing principle of the entire program, but rather as a deliberately embedded small-group format within broader undergraduate or preclinical teaching structures. This makes individual PBL sessions important curricular spaces in which selected biomedical or clinically oriented content can be transformed into problem-oriented, student-centered learning.

Yet the promise of PBL is not automatically realized once students are placed around a problem. Its effectiveness depends on whether the learning environment generates sufficient cognitive, emotional, and social activation to sustain the very processes PBL is designed to promote: elaboration, motivation, collaborative reasoning, and self-directed inquiry. When such activation remains low, PBL may remain structurally present but pedagogically underpowered, limiting the extent to which its intended learning processes are expressed across groups, sessions, and individual learners^8^. This raises an important question for learning research: what kinds of learner states are actually generated within PBL environments, and how do these states relate to engagement, attention, and learning-related behavior?

Learning does not occur in a physiologically neutral state. Educational situations are accompanied by emotional and autonomic dynamics that shape attention, engagement, knowledge retrieval, and behavioral regulation^9,10^. Achievement emotions such as enjoyment, anxiety, and boredom are closely linked to motivation, cognitive resources, self-regulated learning, and academic performance, indicating that the emotional tone of a learning environment is functionally relevant for learning^11,12^. At the neurobiological level, arousal-related systems provide a mechanistic basis for this relationship: sympathetic-adrenomedullary and hypothalamic-pituitary-adrenal activation modulate catecholaminergic and glucocorticoid signaling, thereby influencing amygdala, hippocampal, prefrontal, and striatal networks involved in attention, memory, and cognitive control^13–15^. From this perspective, autonomic regulation provides a peripheral window into how learners adapt to the demands of an educational situation^16^. Sympathetic activation and parasympathetic withdrawal reflect the mobilization of bodily resources, while vagally mediated heart rate variability is closely linked to top-down regulatory capacity, cognitive control, and flexible adaptation to changing task demands^17,18^. Importantly, however, the effects of stress and arousal are not linear. Moderate, task-relevant, and temporally aligned arousal may support attentional mobilization and memory formation, whereas excessive or prolonged stress can impair retrieval, cognitive control, and performance^19–21^. Physiological arousal in learning environments should therefore not be interpreted as inherently beneficial or harmful, but as a context-dependent marker of engagement whose educational meaning depends on intensity, timing, and subjective appraisal. Accordingly, throughout the present study, reduced HRV is interpreted as an indicator of autonomic activation or mobilization, whereas the terms stress and anxiety refer to the corresponding subjective experiences. Neither physiological activation nor subjective stress alone establishes whether the resulting learning state was educationally adaptive or maladaptive. If arousal is context-dependent, it can also be pedagogically designed. In protected learning environments, moderate assessment-like activation may be used strategically to increase attention, accountability, and emotional involvement without converting the situation into a high-stakes threat. Examination simulation offers a concrete mechanism for this purpose. Previous psychophysiological work has shown that simulated oral examination formats, both lecturer-led and peer-led, can evoke measurable physiological arousal in educational settings^22^. Applied to PBL, such simulation may act as a controlled perturbation of a familiar learning routine: it preserves the collaborative, student-centered structure of PBL while adding assessment-like salience that may counteract habituation, underarousal, and reduced engagement.

Against this background, the present study investigated whether implementing a peer-led examination simulation into a regular PBL session changes the emotional and autonomic state architecture of medical students. The present study was not designed to evaluate the comparative instructional effectiveness of the two formats or their effects on knowledge acquisition, retention, or transfer. Instead, it focused on the autonomic and emotional embedding of the learning environment itself: that is, on how an assessment-like, socially embedded instructional episode was associated with physiological regulation, achievement-related emotions, perceived stress, and motivational appraisal while learning unfolded. We therefore conceptualized peer-led examination simulation not simply as an assessment rehearsal, but as a controlled didactic perturbation of PBL designed to increase assessment-like salience while preserving the collaborative and student-led character of the learning environment.

To capture this learning-related state profile, we combined heart rate variability as a marker of autonomic regulation with psychological measures of social interaction anxiety, perceived stress, anxiety, boredom, enjoyment, motivation, self-rated performance, and post-session appraisal. This multimodal approach allowed us to examine whether peer-led examination simulation elicited HRV-based evidence of physiological arousal, whether this arousal co-occurred with systematic changes in achievement-related emotional and motivational states, and whether individual differences in social-evaluative sensitivity modulated students’ affective and autonomic responses. Finally, we explored psychophysiological coupling between emotional activation and vagally mediated HRV withdrawal to test whether subjective anxiety and autonomic regulation converged within the same instructional episode.

## Results

Regular PBL was assessed on 24 November 2023 and PBL with peer-led examination simulation on 15 December 2023, three weeks later; both sessions began at 14:00 and lasted 120 minutes. In each condition, HRV was analyzed in two consecutive 5-minute windows covering minutes 0–5 and 5–10 of each participant’s active performance episode. A separate 10-minute seated resting reference, also divided into two 5-minute windows, was obtained at the end of the first session after case work and introduction of the next curricular case.

### Overview of the analytic structure

The analyses followed the repeated-measures structure of the study. Heart rate variability (HRV) outcomes were analyzed across resting baseline, regular problem-based learning (PBL), and PBL with peer-led examination simulation. Psychological outcomes were analyzed as within-person contrasts between regular PBL and PBL with examination simulation. For HRV, each participant contributed two 5-minute intervals per learning condition, allowing both condition-level paired comparisons and interval-level mixed-effects modeling. Descriptive statistics for HRV parameters are presented in Table 1; descriptive statistics for psychological measures and post-session appraisal are presented in Table 2.

**Table 1:** Descriptive statistics of heart rate variability parameters across baseline, regular PBL, and PBL with peer-led examination simulation

Marker	Phase	Interval	*n*	M (SD)	Mdn [IQR]
RMSSD	Baseline	1	42	48.48 (25.83)	39.52 [28.89]
RMSSD	Baseline	2	42	44.01 (22.03)	39.64 [29.79]
RMSSD	Baseline	Total	42	46.24 (23.51)	41.02 [28.09]
RMSSD	Regular PBL	1	42	26.75 (13.61)	24.52 [19.38]
RMSSD	Regular PBL	2	42	28.83 (15.05)	26.92 [17.43]
RMSSD	Regular PBL	Total	42	27.79 (13.88)	25.99 [16.74]
RMSSD	PBL + Exam Simulation	1	42	21.66 (12.62)	19.67 [16.82]
RMSSD	PBL + Exam Simulation	2	42	23.53 (16.06)	23.79 [14.16]
RMSSD	PBL + Exam Simulation	Total	42	22.59 (13.81)	20.81 [15.69]
pNN50	Baseline	1	42	24.17 (19.38)	17.90 [33.24]
pNN50	Baseline	2	42	22.65 (18.13)	19.12 [33.06]
pNN50	Baseline	Total	42	23.41 (18.35)	19.09 [33.14]
pNN50	Regular PBL	1	42	7.76 (8.44)	4.58 [10.58]
pNN50	Regular PBL	2	42	9.96 (11.42)	6.90 [12.73]
pNN50	Regular PBL	Total	42	8.86 (9.54)	6.01 [11.45]
pNN50	PBL + Exam Simulation	1	42	5.40 (6.47)	3.36 [6.35]
pNN50	PBL + Exam Simulation	2	42	6.61 (10.13)	4.57 [6.46]
pNN50	PBL + Exam Simulation	Total	42	6.00 (8.00)	3.50 [6.72]
SDNN	Baseline	1	42	54.37 (19.35)	51.30 [29.60]
SDNN	Baseline	2	42	51.28 (16.77)	47.62 [22.33]
SDNN	Baseline	Total	42	52.82 (17.55)	48.72 [25.94]
SDNN	Regular PBL	1	42	39.05 (15.52)	36.53 [20.25]
SDNN	Regular PBL	2	42	41.22 (14.47)	38.14 [21.26]
SDNN	Regular PBL	Total	42	40.14 (14.40)	38.24 [22.33]
SDNN	PBL + Exam Simulation	1	42	34.63 (14.18)	32.17 [21.06]
SDNN	PBL + Exam Simulation	2	42	35.98 (14.54)	35.59 [18.64]
SDNN	PBL + Exam Simulation	Total	42	35.31 (13.81)	34.92 [20.45]
LF/HF ratio	Baseline	1	42	2.53 (2.46)	1.85 [2.42]
LF/HF ratio	Baseline	2	42	3.04 (2.76)	2.54 [3.01]
LF/HF ratio	Baseline	Total	42	2.78 (2.45)	2.31 [2.96]
LF/HF ratio	Regular PBL	1	42	3.93 (2.20)	3.66 [3.24]
LF/HF ratio	Regular PBL	2	42	4.19 (2.71)	3.77 [3.52]
LF/HF ratio	Regular PBL	Total	42	4.06 (2.24)	4.00 [2.88]
LF/HF ratio	PBL + Exam Simulation	1	42	4.83 (3.79)	3.80 [4.12]
LF/HF ratio	PBL + Exam Simulation	2	42	4.36 (2.57)	4.22 [3.42]
LF/HF ratio	PBL + Exam Simulation	Total	42	4.59 (2.80)	4.03 [3.17]

Values are reported on the original metric for interpretability. RMSSD and SDNN are given in ms; pNN50 is given in percent; LF/HF ratio is unitless. Total values represent the mean of the two 5-minute intervals.

**Table 2:** Descriptive statistics of psychological measures and post-session appraisal across regular PBL and PBL with peer-led examination simulation

Variable	Condition	Scale	*n*	M (SD)	Mdn [IQR]
Social Interaction Anxiety (SIAS sum)	Pre/baseline trait	Sum score	42	44.95 (11.33)	44.00 [17.75]
Enjoyment	Regular PBL	Scale mean	42	2.37 (0.79)	2.25 [1.25]
Enjoyment	PBL + Exam Simulation	Scale mean	42	2.45 (0.74)	2.25 [1.00]
Anxiety	Regular PBL	Scale mean	42	1.52 (0.59)	1.38 [0.75]
Anxiety	PBL + Exam Simulation	Scale mean	42	2.00 (0.91)	2.00 [0.94]
State anxiety	Regular PBL	VAS	42	3.67 (2.67)	3.00 [5.00]
State anxiety	PBL + Exam Simulation	VAS	42	4.86 (2.50)	5.00 [4.00]
Boredom	Regular PBL	Scale mean	42	2.52 (0.96)	2.38 [1.19]
Boredom	PBL + Exam Simulation	Scale mean	42	2.35 (0.83)	2.12 [1.19]
State boredom	Regular PBL	VAS	42	4.14 (2.82)	4.00 [5.75]
State boredom	PBL + Exam Simulation	VAS	42	2.31 (1.81)	2.00 [1.75]
Motivation	Regular PBL	VAS	42	5.49 (2.10)	5.75 [2.88]
Motivation	PBL + Exam Simulation	VAS	42	5.86 (1.85)	6.00 [2.56]
Perceived stress	Regular PBL	VAS	42	4.62 (2.39)	5.00 [3.75]
Perceived stress	PBL + Exam Simulation	VAS	42	6.31 (2.28)	6.00 [3.00]
Self-confidence	Regular PBL	VAS	42	6.50 (2.12)	6.00 [3.00]
Self-confidence	PBL + Exam Simulation	VAS	42	5.67 (2.31)	5.00 [4.00]
Self-rated performance	Regular PBL	VAS	42	6.24 (1.72)	7.00 [2.00]
Self-rated performance	PBL + Exam Simulation	VAS	42	6.02 (1.76)	6.00 [2.00]
Benefited from Exam Format	Post only	1-5 response	42	3.71 (1.37)	4.00 [2.00]
Benefited from Regular Format	Post only	1-5 response	42	2.64 (1.25)	3.00 [1.00]
Exam Format preferred	Post only	1-5 response	42	3.60 (1.17)	4.00 [1.75]
Regular Format preferred	Post only	1-5 response	42	2.07 (1.18)	2.00 [2.00]
Exam Format was an adequate alternative	Post only	1-5 response	42	3.57 (1.25)	4.00 [2.00]

Only variables reported in figures or model summaries are shown. Scale means are item means; VAS variables are visual analog scale ratings; post-only items were rated from 1 to 5.

To describe condition-related change, within-person differences were defined as

$${\Delta }_{E-R}={X}_{E}-{X}_{R}$$

where ${X}_{E}$ denotes the value in the examination-simulation condition and ${X}_{R}$ the value in regular PBL. For the exploratory coupling analysis, additional RMSSD withdrawal was defined as

$${W}_{RMSSD}=\ln({RMSSD}_{R})-\ln({RMSSD}_{E})$$

such that higher values indicate stronger additional vagally mediated HRV withdrawal during examination simulation relative to regular PBL.

### Peer-led examination simulation induced additional HRV withdrawal beyond regular PBL

Across total HRV values, both learning conditions were characterized by lower HRV relative to resting baseline. RMSSD decreased from baseline (M = 46.24 ms, SD = 23.51) to regular PBL (M = 27.79 ms, SD = 13.88) and was lowest during PBL with peer-led examination simulation (M = 22.59 ms, SD = 13.81). The same graded pattern was observed for SDNN, which decreased from baseline (M = 52.82 ms, SD = 17.55) to regular PBL (M = 40.14 ms, SD = 14.40) and was further reduced during examination simulation (M = 35.31 ms, SD = 13.81). pNN50 showed a comparable reduction from baseline (M = 23.41%, SD = 18.35) to regular PBL (M = 8.86%, SD = 9.54) and examination simulation (M = 6.00%, SD = 8.00). In contrast, LF/HF increased from baseline (M = 2.78, SD = 2.45) to regular PBL (M = 4.06, SD = 2.24) and examination simulation (M = 4.59, SD = 2.80), but showed no robust additional increase during examination simulation.

Paired within-person comparisons confirmed this autonomic response pattern after correction for multiple testing. Log-RMSSD was lower during regular PBL than during the resting reference, Δ = −0.51, 95% CI [−0.61, −0.40], t(41) = − 9.81, *p* < 0.001, *q* < 0.001, dz = −1.51. Critically, log-RMSSD was further reduced during examination simulation compared with regular PBL, Δ = −0.27, 95% CI [−0.41, −0.13], t(41) = − 3.89, *p* < 0.001, q = 0.001, dz = −0.60. Log-SDNN showed the same condition pattern, Δ = −0.15, 95% CI [−0.24, −0.05], t(41) = − 3.10, *p* = 0.003, *q* = 0.007, dz = −0.48. pNN50 was also lower during examination simulation, Δ = −2.86 percentage points, 95% CI [−5.01, −0.71], t(41) = −2.69, *p* = 0.010, *q* = 0.014, dz = −0.41. LF/HF did not differ reliably between the two learning conditions, Δ = 0.09 on the log scale, t(41) = 0.96, *p* = 0.340, *q* = 0.340. There was no evidence that the condition effect differed between the first and second 5-minute intervals for RMSSD, β = −0.029, *p* = 0.855, SDNN, β = −0.090, *p* = 0.584, pNN50, β = −0.105, *p* = 0.541, or LF/HF, β = −0.209, *p* = 0.321. Thus, the observed condition pattern was not detectably restricted to either the initial or later half of the performance episode.

To account for the interval-level structure of the physiological data, linear mixed-effects models were fitted for each HRV marker. The models included examination-simulation condition, the corresponding resting-reference HRV value, measurement interval, standardized SIAS score, and a condition × interval interaction as fixed effects, together with a participant-specific random intercept. This specification allowed us to estimate the examination-simulation effect while accounting for individual resting autonomic regulation, repeated interval measurements, and interindividual differences in social interaction anxiety, and to test whether the condition effect differed between the first and second 5-minute intervals.

**Fig. 1:**
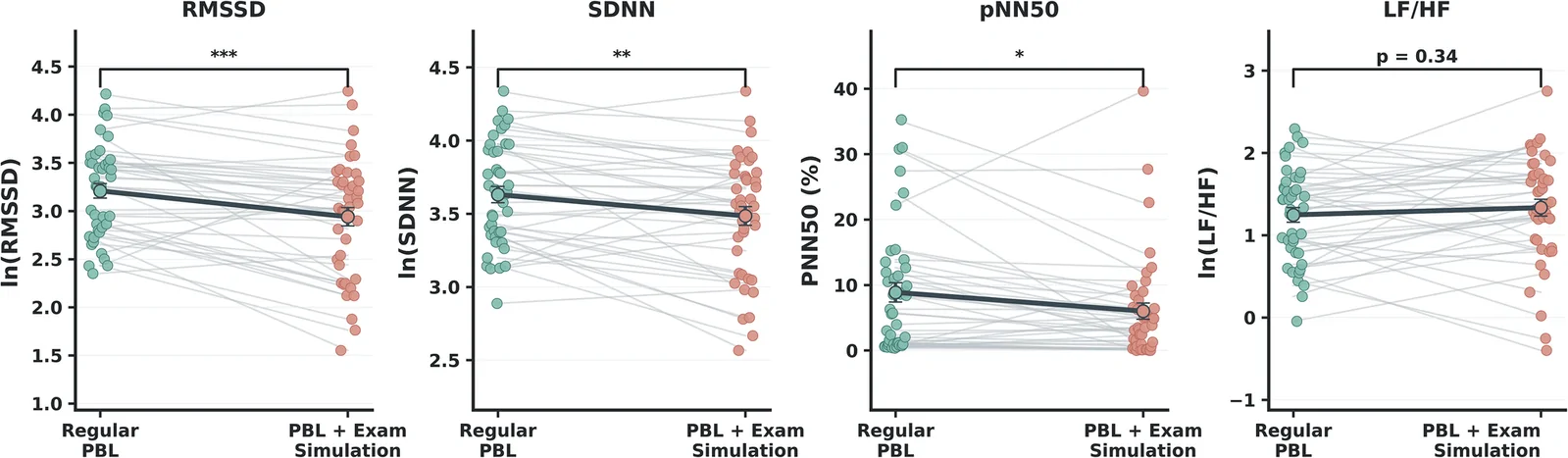
Heart rate variability response to peer-led examination simulation within problem-based learning. Paired within-person comparisons of HRV parameters during regular PBL and PBL with peer-led examination simulation. RMSSD, SDNN, and pNN50 were lower during the examination-simulation condition, indicating stronger vagally mediated HRV withdrawal and reduced overall variability during the assessment-like learning phase. LF/HF showed no reliable difference between conditions. Dots represent individual participants; connecting lines indicate within-person changes; group estimates are shown as mean values with confidence intervals. Asterisks indicate *p < 0.05, **p < 0.01, and ***p < 0.001.

The interval-level models provided corrected evidence for lower standardized RMSSD, β = −0.448, 95% CI [−0.666, −0.230], *p* < 0.001, *q* < 0.001, and lower SDNN, β = −0.312, 95% CI [−0.540, −0.085], *p* = 0.007, *q* = .014, during examination simulation. The corresponding pNN50 coefficient was negative at the unadjusted level, β = −0.253, 95% CI [−0.491, −0.014], *p* = 0.038, but did not remain below the false-discovery-rate threshold, q = 0.0503. No reliable condition effect was observed for LF/HF, β = 0.208, 95% CI [−0.083, 0.500], *p* = 0.161, *q* = 0.161. Thus, the interval-level robustness analyses most clearly supported the examination-simulation effect for RMSSD and SDNN (Fig. 1).

BMI was available for all 42 participants (M = 23.17, SD = 3.34, range 18.38–33.30 kg/m²). Additional BMI adjustment left the condition estimates unchanged at the reported precision: RMSSD (β = −0.448, 95% CI [−0.666, −0.230], q < 0.001) and SDNN (β = −0.312, 95% CI [−0.540, −0.085], *q* = 0.014) remained significant, whereas pNN50 (*q* = 0.0503) and LF/HF (*q* = 0.161) did not meet the FDR-adjusted threshold.

### Peer-led examination simulation shifted the achievement-emotional profile without reducing self-rated performance

The examination simulation produced a differentiated achievement-emotional response profile. Anxiety on the multi-item achievement-emotion scale increased from regular PBL (M = 1.52, SD = 0.59) to PBL with examination simulation (M = 2.00, SD = 0.91), Δ = 0.48, 95% CI [0.23, 0.74], t(41) = 3.81, *p* < 0.001, dz = 0.59. State anxiety assessed by the visual analog scale also increased during examination simulation (M = 4.86, SD = 2.50) compared with regular PBL (M = 3.67, SD = 2.67), Δ = 1.19, 95% CI [0.16, 2.22], t(41) = 2.34, *p* = 0.024, dz = 0.36. Perceived stress was higher during examination simulation (M = 6.31, SD = 2.28) than during regular PBL (M = 4.62, SD = 2.39), Δ = 1.69, 95% CI [0.76, 2.63], t(41) = 3.65, *p* < 0.001, dz = 0.56.

In parallel, state boredom was lower during examination simulation (M = 2.31, SD = 1.81) than during regular PBL (M = 4.14, SD = 2.82), Δ = −1.83, 95% CI [-2.83, −0.84], t(41) = −3.71, *p* < 0.001, dz = −0.57. Enjoyment and self-rated performance did not show reliable paired differences between conditions (all *p* ≥ 0.143). Self-confidence was lower during examination simulation (M = 5.67, SD = 2.31) than during regular PBL (M = 6.50, SD = 2.12), Δ = −0.83, 95% CI [-1.54, −0.12], t(41) = -2.36, *p* = 0.023, dz = −0.36.

**Fig. 2:**
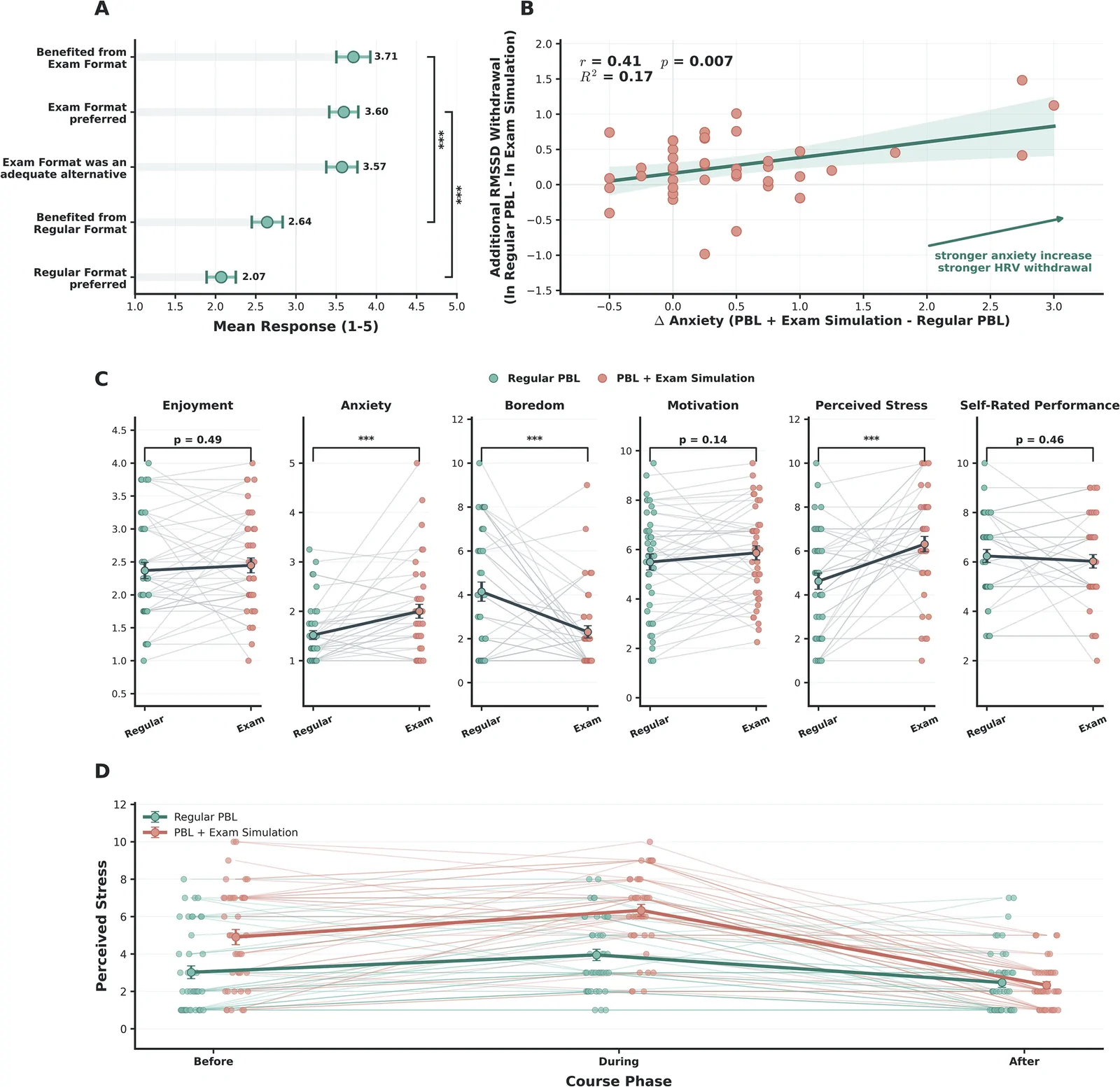
Post-session appraisal, achievement-emotional responses, psychophysiological coupling, and temporal stress profiles across regular PBL and PBL with peer-led examination simulation. **A** Post-session ratings of perceived benefit, format preference, and the adequacy of examination simulation as an alternative to regular PBL. Points represent means; error bars indicate 95% confidence intervals; brackets indicate paired within-person contrasts. **B** Exploratory association between the condition-related increase in achievement anxiety and additional RMSSD withdrawal. Points represent participants; the regression line and shaded area indicate the fitted association and its 95% confidence interval. Greater increases in anxiety were associated with stronger RMSSD withdrawal (r = 0.41, *p* = 0.007, R² = 0.17). **C** Paired within-person comparisons of enjoyment, anxiety, boredom, motivation, perceived stress, and self-rated performance. Individual values are connected across conditions; highlighted points and error bars represent means and 95% confidence intervals. Examination simulation was associated with higher anxiety and perceived stress and lower boredom, whereas enjoyment, motivation, and self-rated performance did not differ reliably. **D** Perceived stress before, during, and after each learning condition. Thin lines show individual trajectories; highlighted lines and error bars represent group means and 95% confidence intervals. PBL problem-based learning, RMSSD root mean square of successive differences. Asterisks indicate *p < 0.05, **p < 0.01, and ***p < 0.001.

Post-session appraisal favored the examination-simulation format. Students reported greater perceived benefit from the examination format (M = 3.71, SD = 1.37) than from the regular format (M = 2.64, SD = 1.25), mean difference = 1.07, 95% CI [0.57, 1.57], t(41) = 4.30, *p* < 0.001, dz = 0.66. They also preferred the examination format more strongly than the regular format (M = 3.60, SD = 1.17 vs. M = 2.07, SD = 1.18), mean difference = 1.52, 95% CI [0.88, 2.17], t(41) = 4.79, *p* < 0.001, dz = 0.74. The examination format was additionally rated as an adequate alternative to the regular format (M = 3.57, SD = 1.25). Together, these findings indicate that the examination simulation increased achievement-related anxiety and perceived stress while reducing boredom; enjoyment and self-rated performance did not differ reliably between conditions (Fig. 2A, C).

### Exploratory convergent-validity analysis

The single-item anxiety rating was positively associated with the corresponding multi-item anxiety score during regular PBL, ρ = 0.36, 95% CI [0.04, .65], *p* = 0.019, *q* = 0.025, and during the examination simulation, *ρ* = 0.53, 95% CI [0.24, 0.75], *p* < 0.001, *q* = 0.001. For boredom, convergence was observed during regular PBL, *ρ* = 0.39, 95% CI [0.09, 0.67], *p* = 0.010, *q* = 0.020, but not during the examination simulation, *ρ* = 0.12, 95% CI [−0.20, 0.42], *p* = 0.470, *q* = 0.470. Pearson sensitivity analyses yielded the same overall interpretation. Thus, convergence was evident for anxiety and partially evident for boredom, but the single-item and multi-item measures were not interchangeable.

### Perceived stress showed anticipatory and in-situ activation but no sustained post-session elevation

Perceived stress ratings across the session provided a temporal manipulation check for the examination-simulation condition. Before the session, stress was already higher in the examination-simulation condition (M = 4.90, SD = 2.63) than before regular PBL (M = 3.02, SD = 2.23). During the intervention, stress increased to M = 6.33 (SD = 2.09) in the examination-simulation condition, compared with M = 3.95 (SD = 1.96) during regular PBL. After the session, stress ratings converged at low levels, with similar values after examination simulation (M = 2.33, SD = 1.22) and regular PBL (M = 2.48, SD = 1.71).

To model the temporal stress trajectory, perceived stress was analyzed using a categorical mixed-effects model:

$${S}_{{it}}= 	 {\beta }_{0}+{\beta }_{1}{E}_{i}+{\beta }_{2}{T}_{t,{during}}+{\beta }_{3}{T}_{t,{post}}+{\beta }_{4}\left({E}_{i}\times \,{T}_{t,{during}}\right) \\ 	 +{\beta }_{5}\left({E}_{i}\times {T}_{t,{post}}\right)+{u}_{i}+{\varepsilon }_{{it}}$$

where ${S}_{{it}}$ denotes standardized perceived stress for participant i at time point t, ${E}_{i}$ denotes the examination-simulation condition, ${T}_{{during}}$ and ${T}_{{post}}$ denote dummy indicators for the during-session and post-session time points, with pre-session serving as the reference category, ${u}_{i}$ is the participant-specific random intercept, and ${\varepsilon }_{{it}}$ is the residual error term.

The model reflected the descriptive stress profile. Examination simulation was associated with higher standardized stress at the pre-session reference point, β = 0.765, 95% CI [0.469, 1.062], *p* < 0.001. Stress increased from pre-session to intervention during regular PBL, β = 0.378, 95% CI [0.082, 0.674], *p* = 0.012. In the examination-simulation condition, stress showed a pronounced post-session reduction relative to its elevated pre-session level, condition × post-session β = −0.823, 95% CI [−1.242, −0.404], *p* < 0.001. SIAS did not reliably moderate the categorical stress trajectory terms (all interaction *p* ≥ 0.373). Thus, the subjective stress trajectory was consistent with anticipatory stress, further elevation during the simulation, and subsequent recovery, with descriptively low stress ratings in both conditions at the post-session assessment (Fig. 2D).

### Social interaction anxiety marked psychological vulnerability but did not substantially moderate the examination-simulation effect

Exploratory mixed models were used to examine whether social interaction anxiety shaped students’ responses to peer-led examination simulation. For standardized psychological outcomes, the core model was

$${Y}_{{ij}}={\beta }_{0}+{\beta }_{1}{E}_{{ij}}+{\beta }_{2}{SIA}{S}_{z,i}+{\beta }_{3}\left({E}_{{ij}}\times \,{SIA}{S}_{z,i}\right)+{u}_{i}+{\varepsilon }_{{ij}}$$

where ${Y}_{{ij}}$ denotes the standardized psychological outcome for participant i at observation j, ${E}_{{ij}}$ denotes the examination-simulation indicator, ${SIA}{S}_{z,i}$ denotes the standardized SIAS score of participant i, ${u}_{i}$ is the participant-specific random intercept, and ${\varepsilon }_{{ij}}$ is the residual error term.

Across models, examination simulation remained a robust predictor of higher perceived stress and anxiety. In the perceived-stress model, which additionally adjusted for RMSSD change from baseline, examination simulation predicted higher standardized stress, β = 0.748, SE = 0.195, 95% CI [0.365, 1.131], *p* < 0.001. SIAS also predicted higher stress, β = 0.307, SE = 0.138, 95% CI [0.037, 0.577], *p* = 0.026, whereas the condition × SIAS interaction was negligible, β = −0.037, *p* = 0.841. RMSSD change from baseline was not a reliable main-effect predictor in this model, β = 0.103, *p* = 0.318.

The same pattern was observed for anxiety-related outcomes. Examination simulation predicted higher standardized anxiety on the achievement-emotion scale, β = 0.602, 95% CI [0.296, 0.907], *p* < 0.001, and higher state anxiety, β = 0.450, 95% CI [0.078, 0.822], *p* = 0.018. SIAS was associated with higher state anxiety, β = 0.370, 95% CI [0.094, 0.646], *p* = 0.009, and with lower self-confidence, β = −0.491, 95% CI [−0.747, −0.235], *p* < 0.001. Examination simulation also predicted lower self-confidence, β = −0.371, 95% CI [−0.675, −0.067], *p* = 0.017.

For autonomic regulation, examination simulation predicted lower standardized log-RMSSD, β = −0.469, 95% CI [−0.702, −0.236], *p* < 0.001. Neither SIAS, β = 0.207, *p* = 0.158, nor the condition × SIAS interaction, β = 0.034, *p* = 0.777, explained additional RMSSD variance. Across outcomes, SIAS therefore appeared to function primarily as a general psychological vulnerability marker rather than as a strong moderator of the examination-simulation effect. The model-based estimates are summarized in Table 3.

**Table 3:** Model-based estimates of the emotional and autonomic response profile induced by peer-led examination simulation

Analysis/outcome	Key predictor	Estimate	SE	95% CI	*p* value	Interpretation
**Perceived stress**	condition_exam	β = 0.748	0.195	[0.365, 1.131]	*p* < 0.001	Exam simulation predicted higher perceived stress.
	SIAS_z	β = 0.307	0.138	[0.037, 0.577]	*p* = 0.026	Higher SIAS predicted generally higher stress.
	condition_exam × SIAS_z	β = −0.037	0.187	[−0.404, 0.329]	*p* = 0.841	SIAS did not moderate the stress effect.
**Anxiety**	condition_exam	β = 0.602	0.156	[0.296, 0.907]	*p* < 0.001	Exam simulation predicted higher anxiety.
	SIAS_z	β = 0.325	0.139	[0.052, 0.598]	*p* = 0.020	Higher SIAS predicted generally higher anxiety.
	condition_exam × SIAS_z	β = −0.009	0.158	[−0.318, 0.301]	*p* = 0.955	SIAS did not amplify the exam effect.
**Anxiety**	condition_exam	β = 0.450	0.190	[0.078, 0.822]	*p* = 0.018	Exam simulation predicted higher state anxiety.
	SIAS_z	β = 0.370	0.141	[0.094, 0.646]	*p* = 0.009	SIAS marked general psychological vulnerability.
**Self-confidence**	condition_exam	β = −0.371	0.155	[−0.675, −0.067]	*p* = 0.017	Exam simulation predicted lower self-confidence.
	SIAS_z	β = −0.491	0.131	[−0.747, −0.235]	*p* < 0.001	Higher SIAS predicted lower self-confidence.
**RMSSD**	condition_exam	β = −0.469	0.119	[−0.702, −0.236]	*p* < 0.001	Exam simulation predicted lower RMSSD.
	condition_exam × SIAS_z	β = 0.034	0.120	[−0.202, 0.270]	*p* = 0.777	SIAS did not moderate RMSSD differences.
**Psychophysiological coupling**	condition_exam × RMSSD_change_z	β = 0.477	0.207	[0.071, 0.884]	*p* = 0.021	Stress-RMSSD coupling differed between conditions.

### Anxiety increases were coupled with additional RMSSD withdrawal

Exploratory psychophysiological coupling analyses tested whether stronger achievement-emotional activation was associated with stronger autonomic withdrawal during examination simulation. The primary coupling analysis related the change in achievement-related anxiety to additional RMSSD withdrawal:

$${W}_{{RMSSD}}=\alpha +\beta {Delta }_{{Anxiety}}+\varepsilon$$

Participants with larger increases in anxiety from regular PBL to examination simulation showed stronger additional RMSSD withdrawal, r = 0.41, *p* = 0.007, R² = 0.17. In the corresponding simple regression, each one-point increase in Δ-anxiety was associated with an additional 0.22 log-unit shift in RMSSD withdrawal. This association is shown in Fig. 2B.

A complementary mixed model further examined whether the association between perceived stress and RMSSD change differed by condition:

$${S}_{{ij}}={\beta }_{0}+{\beta }_{1}{E}_{{ij}}+{\beta }_{2}{SIA}{S}_{z,i}+{\beta }_{3}{C}_{{ij}}^{z}+{\beta }_{4}\left({E}_{{ij}}\times \,{C}_{{ij}}^{z}\right)+{u}_{i}+{\varepsilon }_{{ij}}$$

where ${S}_{{ij}}$ denotes standardized perceived stress and ${C}_{{ij}}^{z}$ denotes standardized RMSSD change from baseline. The condition × RMSSD-change term was positive, β = 0.477, SE = 0.207, 95% CI [0.071, 0.884], *p* = 0.021, indicating that the stress-RMSSD association differed between regular PBL and examination simulation. Because these analyses were exploratory, they should be interpreted as evidence for partial psychophysiological convergence rather than as confirmatory tests of causal coupling. Overall, the coupling findings suggest that the examination-simulation condition linked subjective achievement-emotional activation with vagally mediated autonomic regulation.

## Discussion

The present study shows that embedding a peer-led examination simulation into a regular PBL session produced a distinct psychophysiological and emotional activation profile. Compared with regular PBL, the examination-simulation condition was associated with stronger reductions in HRV, reflected by lower RMSSD, SDNN, and pNN50 values, whereas LF/HF did not differ reliably between the two learning conditions. This pattern was replicated in interval-level mixed models adjusting for baseline HRV and measurement interval, supporting the robustness of the examination-simulation effect for vagally mediated and overall HRV indices. Psychologically, the simulation condition elicited higher state anxiety and perceived stress, while boredom was lower. Importantly, enjoyment and self-rated performance did not show reliable decreases, indicating that the simulation did not produce a broadly negative motivational response. Students additionally reported greater perceived benefit from and stronger preference for the examination format compared with the regular PBL format.

The specific contribution of the present study lies in showing that a familiar, collaborative PBL environment can be shifted into a distinct assessment-like learning state through a low-stakes, peer-led intervention. Despite the absence of grading, unfamiliar examiners, or formal assessment consequences, the simulation elicited additional physiological activation alongside higher anxiety and perceived stress, lower state boredom, and more favorable format appraisal than regular PBL. Importantly, this suggests that assessment-like salience can be deliberately introduced into an established peer-learning environment without abandoning its collaborative character, thereby creating a learning episode in which students must actively retrieve, organize, articulate, and defend previously acquired knowledge under increased performance relevance. Problem-based learning is commonly conceptualized as a learning environment that promotes active knowledge construction, self-directed inquiry, collaborative reasoning, and contextualized application rather than passive reception of information^3^. However, prior work has also emphasized that the educational effectiveness of PBL depends on the quality of its implementation and on whether the learning environment actually activates the cognitive, social, and motivational processes it is intended to stimulate^8^. Against this background, the present findings suggest that peer-led examination simulation may function as a salience-enhancing extension of PBL. By introducing an assessment-like but still formative and student-led element, the simulation shifted the PBL episode from collaborative knowledge exchange alone toward a more performance-relevant context requiring active retrieval, explanation, and defense of knowledge. This interpretation is consistent with assessment-for-learning frameworks, in which assessment-like activities are used to guide and stimulate learning rather than merely to certify performance^23^, and with retrieval-based learning research showing that active recall can strengthen learning more effectively than repeated exposure or passive review^24^.

The HRV profile suggests that peer-led examination simulation increased autonomic mobilization beyond the activation induced by regular PBL. Both learning conditions were characterized by lower HRV relative to baseline, consistent with evidence that HRV provides a peripheral window into mental workload and regulatory effort during cognitively demanding tasks^25,26^. Thus, PBL itself appeared to constitute an active physiological learning state rather than a neutral instructional condition. Critically, paired comparisons showed lower RMSSD, SDNN, and pNN50 during examination simulation than during regular PBL. In the interval-level robustness analyses, the corrected condition effects remained supported for RMSSD and SDNN, whereas the pNN50 coefficient did not remain below the false-discovery-rate threshold. Thus, the simulation effect cannot be reduced to stable individual differences in resting autonomic regulation, but reflects a condition-specific shift in autonomic state. Lower RMSSD and pNN50 indicate stronger withdrawal of vagally mediated HRV, whereas lower SDNN points to reduced overall variability during the assessment-like performance phase^18,27–29^. Within the neurovisceral integration framework, transient vagal withdrawal can be interpreted as a state-dependent adjustment of an integrated central-autonomic regulatory network connecting prefrontal and limbic control regions with vagal and sympathetic outputs to the heart^30^. In the present context, the additional reduction in vagally mediated HRV may therefore index the mobilization of attentional, affective, and social-evaluative regulatory resources required to retrieve, organize, and defend knowledge in an assessment-like peer setting. This interpretation is further supported by the hierarchical extension of the neurovisceral integration model, which conceptualizes cardiac vagal control as the output of a multi-level control architecture integrating brainstem, limbic, interoceptive, and prefrontal systems. Within this framework, higher levels of the hierarchy do not merely respond to current bodily demands, but integrate exteroceptive information, memory, conceptual appraisal, and current goals to regulate autonomic output in anticipation of expected cognitive and social-emotional demands^31^. Thus, the HRV withdrawal observed during the simulation may reflect a state-dependent adjustment of this central-autonomic hierarchy to the predicted and ongoing demands of an assessment-like learning episode. However, HRV withdrawal alone cannot distinguish adaptive task-related mobilization from maladaptive stress; this distinction requires consideration of subjective appraisal, temporal persistence, and educational outcomes.

Achievement-emotion theory conceptualizes learning-related emotions not as peripheral reactions to instruction, but as state variables that shape attention, motivation, cognitive resources, self-regulated learning, and performance-related behavior^11^. Within this framework, emotions differ not only in valence, but also in their activating or deactivating quality. This distinction is important for interpreting the present findings. The peer-led examination simulation increased anxiety and perceived stress compared with regular PBL, indicating that the added assessment-like element raised the emotional demand and perceived situational relevance of the learning episode. Enjoyment, motivation, and self-rated performance did not differ significantly between conditions. Because performance was assessed exclusively through self-report, this finding reflects students’ subjective appraisal and should not be interpreted as evidence of equivalent or objectively preserved performance. At the same time, boredom was reduced, which is relevant because achievement-related boredom is conceptualized as a deactivating emotion arising from low control–value appraisals and has been associated with attention problems, reduced intrinsic motivation, lower effort, weaker elaboration and self-regulation, and poorer academic performance^9,32^. This emotional shift was accompanied by lower RMSSD, pNN50, and SDNN, indicating reduced parasympathetically mediated and overall HRV during the examination-simulation condition. The convergence of reduced boredom and stronger autonomic mobilization suggests that the simulation increased the salience and regulatory demands of the PBL episode, rather than merely inducing nonspecific distress. Because the simulation required students to retrieve, explain, and defend previously prepared knowledge, it is conceptually related to assessment-for-learning and retrieval-practice frameworks. However, the present study did not assess whether these processes translated into improved knowledge, retention, transfer, or objectively evaluated performance. The observed profile indicates greater emotional and autonomic activation during examination simulation, with no statistically significant differences in enjoyment, motivation, or self-rated performance. These findings do not establish an optimal arousal level or improved learning.

The exploratory coupling analyses indicate partial psychophysiological convergence during peer-led examination simulation: stronger increases in anxiety were associated with stronger additional RMSSD withdrawal. This suggests that vagal withdrawal was not merely a nonspecific physiological response, but was linked to learners’ subjective appraisal of the assessment-like situation. Conceptually, this aligns with models of HRV as a marker of stress-related regulatory effort and integrated cognitive-affective control^29,31,33–35^. At the same time, social interaction anxiety showed a selective susceptibility pattern. SIAS predicted higher state anxiety and lower self-confidence, but did not robustly moderate stress ratings, RMSSD, or the temporal stress trajectory. Thus, the simulation appears to have elicited a broadly shared autonomic activation response, while socially more anxious students showed greater vulnerability at the level of subjective appraisal. This distinction is consistent with recent work emphasizing individual variability in subjective–physiological coupling and HRV dynamics during social-evaluative stress^36^.

The study was embedded in an authentic curricular PBL course, strengthening ecological validity while imposing the constraints of a quasi-experimental field design. The learning conditions followed the scheduled curriculum and were neither randomized nor counterbalanced. Although their separation across different sessions excluded immediate same-day carry-over and cumulative within-session fatigue, effects of instructional format cannot be fully separated from session order, curricular progression, or case-specific characteristics. The findings should therefore be interpreted as associations within an authentic curricular comparison rather than as isolated causal effects of the examination-simulation format.

The study involved a single cohort at one medical faculty, limiting population-level generalizability. Although an established seven-step, tutor-facilitated PBL procedure was implemented consistently across groups and sessions, institutional differences in curricular embedding, prior familiarity with PBL, and assessment culture may influence the magnitude and appraisal of the observed state profile. Multicenter studies are therefore needed to examine transferability across curricula and student populations.

The study focused on psychophysiological and achievement-emotional states and did not include objective measures of knowledge acquisition, retention, transfer, or externally rated performance. Self-rated performance should therefore be interpreted as a subjective appraisal rather than as an indicator of actual learning or performance. Consequently, the present data cannot determine whether the observed activation was educationally optimal, adaptive, or maladaptive. Stable motivation, self-rated performance, and positive format appraisal argue against a broadly aversive response but cannot substitute for objective outcomes. Future studies should combine psychophysiological state measures with objective learning outcomes, behavioral observations, and facilitator-based assessments.

The emotion measures also have psychometric limitations. With only 42 independent participants, the study was not designed to support a reliable confirmatory factor analysis of the adapted multi-item measures. Even minor contextual wording changes may affect their measurement properties. Internal consistency and exploratory convergent correlations therefore do not establish factorial validity or measurement invariance across conditions.

The appraisal outcomes were subjective and collected during the initial implementation of the examination-simulation format. Although responses were anonymous and unavailable to course facilitators, the design cannot distinguish the specific contribution of the peer-led interaction from novelty, relief from routine, or Hawthorne-type effects. These findings should therefore be interpreted as reflecting students’ subjective experience of the format rather than its intrinsic pedagogical superiority.

The alternating examinee and peer-examiner roles were deliberately incorporated to preserve core features of PBL, including learner ownership of learning objectives, active questioning, and collaborative discussion. Although HRV was analyzed during participants’ examinee performance, the physiological profile should be interpreted within this deliberately reciprocal learning arrangement rather than attributed exclusively to the examinee role in isolation. This combined format was the intended educational intervention, introducing assessment-like demands while retaining students’ responsibility for shaping the learning process.

Respiration was neither paced nor continuously recorded and could therefore not be statistically controlled. Although both conditions were conducted at the same time of day, in a seated position, and during verbal performance, regular PBL and structured examination questioning likely differed in speech cadence, speaking time, and respiratory timing. The direction of change was consistent across RMSSD, SDNN, and pNN50, although only the RMSSD and SDNN effects remained supported in baseline-adjusted models after multiplicity correction. Together with the concurrent increases in anxiety and perceived stress, this pattern is consistent with broader condition-related psychophysiological activation. However, respiratory and non-respiratory contributions cannot be disentangled, and the HRV reductions cannot be attributed specifically to cardiac parasympathetic withdrawal. Smoking status and physical activity were not recorded, leaving their potential contributions, particularly differences in behavior between sessions, unaccounted for despite the within-participant design and additional BMI adjustment.

Finally, the analyses used two consecutive 5-minute intervals individually anchored to the documented onset of each participant’s active performance phase. This approach enabled standardized participant-specific alignment and the analysis of established short-term HRV windows, but primarily characterized broader condition-related state profiles. Future studies could complement this design with more fine-grained time-stamped annotations of events such as anticipatory waiting, question onset, active retrieval, explanation, peer evaluation, feedback, and recovery. Combining these annotations with respiratory and speech measures, objective learning outcomes, behavioral observations, and complementary physiological markers such as electrodermal activity, pre-ejection period, peripheral temperature, or pupillometry could provide a more differentiated account of autonomic, respiratory, and cognitive-affective dynamics during examination simulation.

Within a structured, tutor-facilitated PBL setting, peer-led examination simulation was associated with a distinct psychophysiological and emotional state profile. By combining repeated-measures HRV assessment, psychological state measures, post-session appraisal, and baseline-adjusted mixed models, the study demonstrates that the examination simulation elicited a coherent activation pattern across autonomic, emotional, and evaluative domains. Compared with regular PBL, the simulation induced additional vagally mediated HRV withdrawal, higher anxiety and perceived stress, and lower boredom, while enjoyment and self-rated performance did not differ reliably between conditions. At the same time, students reported greater perceived benefit from, and stronger preference for, the examination format. These findings indicate that peer-led examination simulation was associated with a more salient, assessment-like psychophysiological and emotional state profile that students appraised more positively than regular PBL. Whether this state profile translates into improved learning, retention, or transfer remains to be determined. More broadly, these findings support the use of psychophysiological measurement to examine how instructional design modulates attention, emotion, and autonomic regulation during authentic medical learning.

## Methods

### Study design and setting

This study used a repeated-measures psychophysiological design embedded in a regular problem-based learning course within the preclinical medical curriculum. At Ruhr University Bochum, PBL is implemented as a recurrent small-group format within a broader integrated medical curriculum. Sessions follow the established seven-step PBL procedure described in the medical education literature, comprising case clarification, activation of prior knowledge, formulation of learning objectives, self-directed study, and subsequent presentation and collaborative discussion^37^. The study compared students’ autonomic and psychological responses across three within-person states: a resting baseline, a regular problem-based learning condition, and an extended problem-based learning condition incorporating a peer-led examination simulation.

The regular PBL condition represented the established course format. Physiological measurements were obtained during step 7 of the PBL cycle, in which students presented, discussed, and integrated the results of their self-directed learning phase within the group. The groups had remained together since the beginning of medical school, and participants had therefore worked regularly with the same peers for more than a year before the study. The examination-simulation condition was implemented as an extension of the same curricular PBL context. In this condition, the regular presentation and discussion phase was replaced by a peer-led oral examination simulation, in which students were questioned by a student-led examination committee on the learning objectives and content of the PBL case. During each session, the onset and duration of every participant’s active performance phase were documented by the research team. These time stamps were subsequently used to extract two consecutive 5-minute HRV intervals from the physiological recordings. Thus, the analyzed segments were selected according to a predefined event-based protocol rather than from arbitrary positions within the broader session. A resting HRV reference was obtained at the end of the first, regular PBL session, after completion of the active case work and introduction of the subsequent curricular case. This timing was chosen to reduce anticipatory activation associated with an upcoming performance episode and to avoid an immediate recovery measurement following the examination simulation. Participants then completed a 10-minute seated resting recording, analyzed as two consecutive 5-minute intervals. Accordingly, this measurement served as a resting physiological reference rather than as a pre-session baseline.

Psychological measures were collected to assess social interaction anxiety, perceived stress, state anxiety, achievement emotions, self-rated performance, and post-session appraisal of the learning formats.

The two learning conditions were conducted during separate, regularly scheduled curricular sessions three weeks apart. PBL sessions were conducted in groups of 10–11 students. Regular PBL was assessed on 24 November 2023, and PBL with peer-led examination simulation was assessed on 15 December 2023. Both sessions began at 14:00, lasted 120 minutes, and involved the same 42 participants. All participants completed the conditions in the same fixed order; condition order was neither randomized nor counterbalanced. The separation of the sessions excluded immediate within-session carry-over and cumulative same-day fatigue, but did not permit instructional-format effects to be fully separated from session order, curricular progression, or other date-related influences. The two sessions addressed distinct but comparably structured preclinical case topics. The two cases were selected before data collection because the teaching team considered them comparable in curricular level, scope, complexity, and expected preparation demands.

The first session was embedded in the metabolism and digestion block and centered on chronic liver disease, using chronic hepatitis B with subsequent liver cirrhosis and portal hypertension as the clinical framework for integrating relevant anatomical, physiological, and biochemical concepts. This session followed the regular PBL format, in which students presented, discussed, and integrated the learning objectives developed during case work. The second session was embedded in the fluid and renal block and centered on renal filtration and fluid homeostasis, using nephrotic syndrome caused by membranous glomerulonephritis, with pronounced proteinuria, hypoalbuminemia, edema, and impaired renal function, as its clinical framework. This session incorporated the peer-led examination simulation. Students developed examination questions from the learning objectives generated during case work. Two representative questions used during the simulation were: “Which structures constitute the glomerular filtration barrier, and how are they organized within the renal corpuscle?” and “Which hydrostatic and oncotic forces determine net filtration pressure in the glomerulus, and how would changes in these forces affect glomerular filtration?” The peer-led examination drew on a broader range of student-generated questions spanning the anatomical, physiological, and biochemical aspects of this curricular case.

The curricular cases were subject to continuous evaluation and iterative revision based on feedback from clinical and preclinical PBL tutors. All participants alternated between examinee and peer-examiner roles. Before the first regular PBL session, trained study personnel explained the study procedures, including participants’ roles as examinees and peer examiners in the subsequent examination simulation, as part of the informed-consent process. Questions were developed from the shared curricular case and jointly defined learning objectives rather than from an externally standardized assessment blueprint. Peer examiners did not assign scores and received no formal assessor training because the simulation was formative and independent of course grading. PBL tutors were trained in the study protocol, and a member of the study organization team attended each session to document participant-specific phase timing, ensure protocol adherence, and support the standardized conduct of data collection. Each examination was followed by immediate formative peer feedback.

### Participants

Forty-two third-semester medical students participated in the study. All participants were enrolled in the preclinical medical curriculum at Ruhr University Bochum and attended the PBL course in which the study was embedded. Participation was voluntary, and all participants provided written informed consent prior to data collection. No participants were excluded from the analyses, and complete physiological and psychological datasets were available for all 42 participants: 23 female students (54.76%) and 19 male students (45.24%). On average, the students in the sample cohort were 20.98 years old (SD = 2.98). Male students had a mean age of 21.47 years (SD = 4.09), whereas female students had a mean age of 20.57 years (SD = 1.53).

### Inclusion criteria

Participants were recruited from the third preclinical semester of the medical curriculum at Ruhr University Bochum. Eligible participants were required to be formally enrolled as medical students and to attend the respective PBL course in which the study was embedded. During recruitment, we aimed to obtain a balanced gender distribution and to include students irrespective of gender identity, religious affiliation, or ethnic background.

### Heart rate variability measurements

Heart rate variability was recorded using the Polar Vantage V2 together with the Polar H10 chest strap sensor (Polar Electro Oy, Kempele, Finland). The Polar H10 was selected because previous validation work has shown high agreement with electrocardiography-derived RR intervals and linear heart rate parameters, supporting its use for HRV assessment in applied research settings^38^. HRV recording was embedded into both learning conditions using a participant-specific, event-anchored measurement protocol. The analyzed segments were not selected freely from the broader 120-minute sessions or according to the resulting HRV profiles. Instead, the onset of each participant’s active performance episode was time-stamped during data collection.

Device-derived beat-to-beat RR interval series were processed using Kubios HRV version 3.4.3 (Kubios Oy, Kuopio, Finland). Threshold-based beat correction was applied at the medium level. RR intervals deviating from a median-filtered local reference beyond a heart-rate-adjusted threshold, nominally corresponding to 0.25 s at a mean heart rate of 60 beats/min, were identified and reconstructed using cubic spline interpolation. The same preprocessing procedure was applied consistently across all participants and conditions. No participant-level exclusions were required following preprocessing, and complete physiological datasets from all 42 participants were retained.

In regular PBL, this episode corresponded to the participant’s presentation and discussion of the assigned learning objective during step 7 of the PBL cycle. In the examination-simulation condition, it corresponded to the participant’s individual 10-minute oral examination conducted by the peer-led examination committee. For both conditions, the performance episode was divided into two consecutive 5-minute intervals, corresponding to minutes 0–5 and 5–10 relative to the onset of the individual performance. Both intervals characterized HRV during active task performance; they did not separately capture anticipatory or recovery phases or resolve the onset of acute physiological stress reactivity. The same extraction rule was applied to all participants and was defined before inspection of the condition-related HRV results. The intervals therefore represented temporally aligned portions of each participant’s active verbal performance rather than fixed clock-time positions within the broader PBL session. Both performance episodes were completed in a seated position and involved verbal presentation or response in front of peers. However, the speech demands were not identical: regular PBL involved presentation and group discussion, whereas the examination simulation involved responding to structured questions. Respiration, speech cadence, and individual speaking time were not recorded and could therefore not be included as physiological covariates. In accordance with the standards proposed by the Task Force of the European Society of Cardiology and the North American Society of Pacing and Electrophysiology^39^, HRV analysis included both time-domain and frequency-domain parameters. Time-domain indices comprised RMSSD, SDNN, and pNN50, while frequency-domain analysis included low-frequency power, high-frequency power, and the LF/HF ratio. Spectral components were derived using a fast Fourier transform algorithm. RMSSD and pNN50 were interpreted primarily as indices of vagally mediated HRV, whereas SDNN was used as a broader marker of overall heart rate variability. The LF/HF ratio was included as an exploratory frequency-domain parameter and interpreted cautiously, given ongoing debate regarding its physiological specificity.

### Psychological measures

Social interaction anxiety was assessed using the Social Interaction Anxiety Scale (SIAS), a validated instrument designed to capture anxiety in social interaction situations^40^. The SIAS was included as a person-level measure of social-evaluative sensitivity that may shape students’ affective and physiological responses during peer-led examination simulation. In the present sample, the SIAS showed high internal consistency (Cronbach’s α = 0.885).

Learning-related emotions were assessed using validated items from the Achievement Emotions Questionnaire, focusing on enjoyment, anxiety, and boredom as central emotional states in academic learning, with only minor contextual wording adaptations where necessary to refer explicitly to the PBL setting; the item content and scale structure were otherwise retained unchanged^41^. These items were selected because achievement emotions are theoretically and empirically linked to motivational engagement, cognitive resource allocation, self-regulated learning, and performance-related appraisal. By including both activating and deactivating emotions, the questionnaire captured whether the peer-led examination simulation produced a differentiated emotional learning profile rather than a nonspecific stress response alone. Internal consistency was good in both learning conditions for enjoyment (regular PBL: α = 0.840; examination simulation: α = 0.850), anxiety (regular PBL: α = 0.804; examination simulation: α = 0.849), and boredom (regular PBL: α = 0.853; examination simulation: α = 0.783). The distinction between trait-like and state achievement emotions depends on the temporal reference of the assessment instructions rather than on the AEQ format itself^41^. To provide an explicitly situation-focused complement to the AEQ-derived measures, additional VAS ratings assessed the intensity of anxiety and boredom experienced during the respective learning episode with minimal additional assessment burden.

In addition, visual analog scales (VAS) were used for brief assessment of situation-specific psychological states and appraisals^42^. Brief ratings of discrete emotions have been used in educational and psychophysiological research, including experience-sampling and testing contexts^43,44^. Complementary validation studies support single-item anxiety assessment through associations with established multi-item measures and, for computerized VAS, sensitivity to situational increases in anxiety^45–47^. In the present study, ratings assessed state anxiety, boredom, perceived stress, motivation, self-confidence, and self-rated performance. Each item was presented with a standardized prompt above a continuous line, along which participants positioned a slider between 1 and 10 to indicate the intensity of the respective state or level of appraisal. Higher scores indicated greater intensity or a higher self-assessed level. Perceived stress was measured before, during, and after each learning condition to characterize the temporal stress profile of regular PBL and PBL with peer-led examination simulation. These assessments captured anticipatory stress before the session, stress experienced during the session, and perceived stress at the post-session recovery assessment. Changes between these assessments characterized the subjective stress trajectory, without resolving the onset or rate of stress reactivity and recovery.

Finally, post-session appraisal items were used to assess students’ perceived educational benefit, format preference, and perceived adequacy of the examination simulation as an alternative or extension to the regular PBL format. Post-session appraisal was completed anonymously. Individual responses were not accessible to course facilitators or teaching staff and had no influence on course participation, grading, or curricular progression. Together, these measures enabled us to characterize not only whether the peer-led examination simulation elicited stress or anxiety, but whether it produced a broader learning-related emotional and evaluative profile.

### Ethical aspects

This research was conducted in accordance with the principles outlined in the Declaration of Helsinki. Approval for the study was obtained from the *Ethics Committee of the Professional School of Education* at Ruhr University Bochum (Reference No. EPSE-2022-004, dated 12.09.2022). The study adhered to all ethical guidelines to ensure the safety, well-being, and confidentiality of all participants. Participation was voluntary, and students were informed before data collection that they could decline participation or withdraw at any time without disadvantage. Participation, withdrawal, examination performance, and questionnaire responses had no influence on course grading, course completion, or curricular progression. Written informed consent was obtained from all participants.

### Statistics

All analyses were based on the repeated-measures structure of the study, with each participant contributing observations from the regular PBL condition, the PBL + examination simulation condition, and the resting baseline where applicable. HRV data were organized at the participant-by-condition-by-interval level, whereas psychological outcomes were analyzed as within-person condition contrasts between regular PBL and PBL + examination simulation. Descriptive statistics are reported as means and standard deviations as well as medians and interquartile ranges.

Analyses were organized into principal, secondary, and exploratory components. This hierarchy was not preregistered. For interpretative organization, the examination-simulation versus regular-PBL contrast for log-RMSSD was treated as the principal physiological outcome because RMSSD most directly represents the vagally mediated component of HRV relevant to the study question. The remaining HRV markers, psychological paired comparisons, post-session appraisal outcomes, and interval-level HRV mixed-model condition coefficients were treated as secondary analyses. SIAS moderation, temporal stress, and psychophysiological coupling analyses were considered exploratory and hypothesis-generating.

For HRV analyses, short-term segments of 5 minutes were used in accordance with established HRV recommendations. Time-domain indices included RMSSD, SDNN, and pNN50; frequency-domain analysis included LF, HF, and the LF/HF ratio. RMSSD, SDNN, and LF/HF were analyzed on the logarithmic scale when required to reduce skewness and improve model assumptions, while descriptive values are reported on the original metric for interpretability. For each participant and condition, interval-level HRV values were retained for mixed-model analyses, and condition-level total values were computed by averaging the two 5-minute intervals. This allowed us to examine both phase-level differences and the robustness of condition effects across repeated physiological segments.

Primary within-person comparisons tested whether regular PBL differed from resting baseline and whether PBL + examination simulation differed from regular PBL. Paired-samples t-tests were used for condition contrasts on the appropriate analysis scale. Effect sizes for paired comparisons are reported as Cohen’s dz, together with mean differences and 95% confidence intervals. This analysis provided a direct estimate of the magnitude of autonomic and psychological change induced by the examination-simulation extension.

To account for the nested structure of the HRV data and to avoid reducing the physiological signal to a single aggregate per participant, we additionally fitted interval-level linear mixed-effects models. These models included participant-specific random intercepts and estimated the effect of examination simulation while adjusting for resting baseline HRV and measurement interval. Measurement interval was coded as the first versus second consecutive 5-minute segment of the participant-specific performance episode. A condition-by-interval interaction was included to examine whether the difference between regular PBL and examination simulation varied between the initial and later halves of the performance episode.

The models additionally included standardized SIAS scores as a person-level covariate. In a sensitivity analysis, all four HRV models were refitted with BMI, calculated as weight in kilograms divided by height in meters squared and centered at the sample mean, as an additional continuous covariate. All other model terms, transformations, and standardization procedures were retained, and both model versions included the same 42 participants. Benjamini–Hochberg correction was applied across the four BMI-adjusted condition coefficients, which represented examination simulation versus regular PBL in the first 5-minute interval.

Separate models were fitted for RMSSD, SDNN, pNN50, and LF/HF. Model predictors were standardized where appropriate, allowing fixed-effect estimates to be interpreted as standardized differences. This modeling strategy was used to test whether the examination-simulation effect remained robust after accounting for individual baseline autonomic regulation and repeated interval measurements.

Psychological outcomes were analyzed using paired within-person comparisons between regular PBL and PBL + examination simulation. For post-session appraisal, paired comparisons examined whether students reported greater perceived benefit from, and preference for, the examination format compared with the regular PBL format.

As an exploratory assessment of convergent validity, the single-item ratings of anxiety and boredom were correlated with the corresponding multi-item achievement-emotion scores separately within each learning condition using Spearman rank correlations. Participant-level bootstrap resampling with 10,000 repetitions was used to estimate 95% confidence intervals. Benjamini–Hochberg correction was applied across the four correlations, and Pearson correlations were calculated as sensitivity analyses.

To examine whether individual differences in social-evaluative sensitivity shaped students’ responses to the peer-led examination simulation, exploratory mixed models included Social Interaction Anxiety Scale scores as a standardized person-level predictor. These models estimated the main effect of condition, the main effect of SIAS, and the condition × SIAS interaction for selected affective, appraisal-related, and autonomic outcomes. This approach allowed us to distinguish between the general effect of the examination simulation and interindividual susceptibility to social-evaluative demands.

Finally, exploratory psychophysiological coupling analyses tested whether changes in subjective emotional activation were associated with changes in autonomic regulation. Specifically, condition-related changes in anxiety were related to additional RMSSD withdrawal from regular PBL to PBL + examination simulation. Complementary regression and mixed-model analyses were used to examine whether subjective anxiety and vagally mediated HRV converged as coordinated markers of the same assessment-like learning state.

For the temporal stress profile, perceived stress ratings obtained before, during, and after each learning condition were analyzed using categorical mixed-effects models with participant-specific random intercepts. Fixed effects included condition, time point, and their interaction, with SIAS included as an additional person-level predictor in exploratory moderation models. This analysis tested whether the examination simulation altered not only the level of perceived stress but also its temporal trajectory across the learning episode.

To address multiplicity, Benjamini–Hochberg false-discovery-rate correction was applied separately within conceptually defined families of related outcomes: HRV comparisons between resting reference and regular PBL, HRV comparisons between examination simulation and regular PBL, interval-level HRV mixed-model condition coefficients, psychological condition comparisons, and paired post-session appraisal comparisons. Adjusted *p*-values are reported as *q*-values. A global false-discovery-rate correction across all secondary non-exploratory tests was additionally conducted as a sensitivity analysis. Exploratory moderation, temporal, and coupling analyses were not included in these confirmatory correction families and are reported with unadjusted *p*-values.

Statistical significance was evaluated using two-sided tests with an alpha level of .05. Emphasis was placed on effect sizes, confidence intervals, and convergence across paired comparisons, interval-level mixed models, and psychophysiological coupling analyses rather than on isolated *p*-values alone.

### Sample-size calculation

Before data collection, the required sample size was estimated for the central within-participant comparison of HRV between learning conditions. The anticipated effect was informed by two preceding empirical studies conducted by our group that demonstrated moderate-to-large condition-related HRV differences in educational learning environments and oral-examination simulations^22,26^. Because the present intervention was embedded within regular PBL and was expected to produce a less pronounced contrast than a stand-alone oral examination simulation, we assumed a conservative standardized within-participant effect of dz=0.60. A two-sided paired-samples test with α = .05 and 95% power indicated a required minimum sample size of 39 participants. We therefore targeted a sample of 42 participants.

### Declaration of generative AI and AI-assisted technologies in the writing process

During manuscript preparation and revision, the authors used ChatGPT (OpenAI) to support language editing of selected passages in the Abstract, Introduction, Methods, Results, and Discussion. Its use was limited to grammar correction, sentence rephrasing, shortening, and improvements in the clarity and coherence of author-directed text. The tool was not treated as an independent source of scientific evidence or as an autonomous decision-maker. All decisions regarding the study design, analyses, selection and verification of results, interpretation, citations, and conclusions were made and critically reviewed by the authors, who take full responsibility for the final manuscript.

## Data Availability

The de-identified data supporting the findings of this study are available from the corresponding author upon reasonable request, subject to the conditions of the ethical approval and applicable data-protection requirements.

## Code availability

Analysis code used to generate the reported statistical results is available from the corresponding author upon reasonable request.
